# Triterpenoid Saponins from *Anemone rivularis* var. *Flore-Minore* and Their Anti-Proliferative Activity on HSC-T6 Cells

**DOI:** 10.3390/molecules23020491

**Published:** 2018-02-23

**Authors:** Xiao-Yang Wang, Hui Gao, Xiao-Jie Xie, Jirimubatu Jurhiin, Mu-Zi-He Zhang, Yan-Ping Zhou, Rui Liu, Meng Ning, Jin Han, Hai-Feng Tang

**Affiliations:** 1 Department of Pharmacy, People’s Liberation Army Institute of Chinese Medicine, Beijing 100039, China; steveplum@sina.com (X.-Y.W.); xiexiaojie1991@163.com (X.-J.X.); zhangmuzihe@163.com (M.-Z.-H.Z.); zhouyp0102@126.com (Y.-P.Z.); liurui198003@yeah.net (R.L.); lemon1572@163.com (M.N.); 2 People's Liberation Army Institute of Organ Transplantation, Beijing 100091, China; gaohui309@163.com; 3 Academy of Mongolian Medicine, Inner Mongolia Medical University, Hohhot 010110, China; jormbut@163.com; 4 Institute of Materia Medica, School of Pharmacy, The Fourth Military Medical University, Xi’an 710032, China

**Keywords:** Triterpenoid saponins, *Anemone rivularis* var. *flore-minore*, Anti-proliferative activity, HSC-T6

## Abstract

Five previously undescribed triterpenoid saponins (**1**–**5**), along with eight known ones (**6**–**13**), were isolated from the whole plants of *Anemone rivularis* var. *flore-minore*. Their structures were clarified by extensive spectroscopic data and chemical evidence. For the first time, the lupane-type saponins (**3** and **12**) were reported from the *Anemone* genus. The anti-proliferative activity of all isolated saponins was evaluated on hepatic stellate cells (HSC-T6). Saponins **12** and **13**, which possess more monosaccharides than the others, displayed potent anti-proliferative activity, with IC_50_ values of 18.21 and 15.56 μM, respectively.

## 1. Introduction

**Figure 1:**
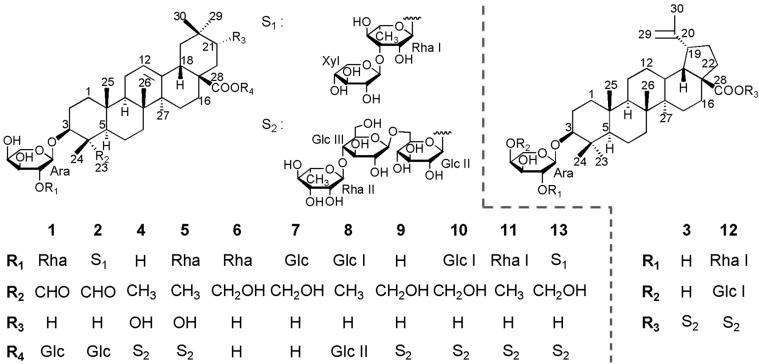
Structures of saponins **1**–**13**.

The genus *Anemone* belongs to the family Ranunculaceae, which consists of about 150 species with a near global distribution. Triterpenoid saponins have been proved to be the main bioactive substances of this genus, which possess potentially useful bioactivities. These bioactivities include antitumor, antibacterial, insect deterrence, and anti-peroxidation, among others [1,2,3,4,5,6,7,8]. For a long time, several species of this genus, such as *A. flaccida*, *A. raddeana*, *A. tomentosa*, *A. anhuiensis*, *A. altaica*, have been used as Chinese traditional medicines. *Anemone rivularis* var. *flore-minore* is widely distributed in western China. The whole plants of *A. rivularis* var. *flore-minore,* named “Poniuqi”, have been used as a folk medicine in Shaanxi Province for the treatment of hepatitis, stranguria, edema, emissions, etc. [9]. As part of our continuing study to explore bioactive natural products from the genus *Anemone* [10,11,12,13,14,15,16,17], we continued the investigation of this plant. In our present study, five new triterpenoid saponins (**1**–**5**), together with eight known ones (**6**–**13**) (Figure 1) were isolated, among which lupane-type saponins (**3** and **12**) were first reported from the *Anemone* genus. Herein, we describe the isolation and structural identification of these saponins, as well as their anti-proliferative activity on HSC-T6 cells.

## 2. Results and Discussion

**Figure 2:**
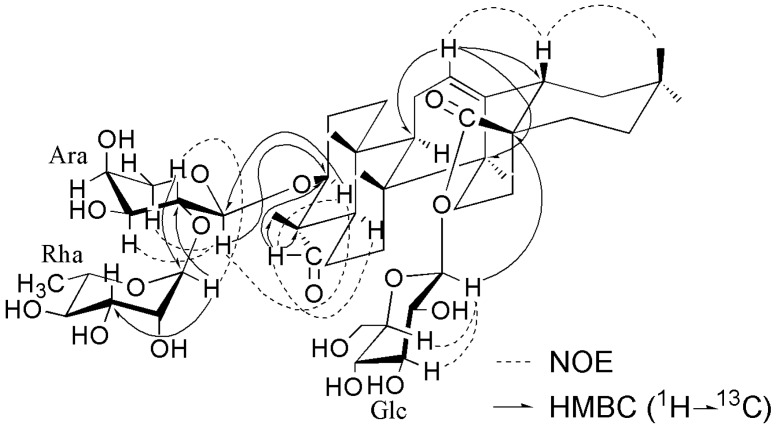
Key NOESY and HMBC correlations for compound **1**.

Saponin **1** was obtained as a white amorphous powder and showed positive results in the Liebermann–Burchard and Molisch tests. Its molecular formula was established as C_47_H_74_O_17_ (*m*/*z* 933.4829 [M + Na]^+^, calcd. for C_47_H_74_O_17_Na^+^, 933.4825) by high resolution electrospray ionization mass spectrometry (HRESIMS). The ^1^H and ^13^C NMR spectra exhibited signals for six tertiary methyl groups at δ_H_ 0.86 (H_3_-29), 0.87 (H_3_-30), 0.89 (H_3_-25), 1.05 (H_3_-25), 1.19 (H_3_-27), and 1.34 (H_3_-24), one olefinic proton signal at δ_H_ 5.41 (1H, br s) with two typical olefinic carbon signals at δ_C_ 122.7 and 144.1, one carbonyl signal at δ_C_ 176.6, and one aldehyde proton signal at δ_H_ 9.65 (1H, s), with the corresponding aldehyde carbon signal at δ_C_ 206.5. These data indicated that **1** was an oleanane-type saponin with one of the aglycone methyl groups substituted by an aldehyde function. The aldehyde function located at C-23 was deduced from the highfield shifts (−6.7 ppm, −8.2 ppm, and −6.9 ppm) exhibited by C-3 (δ_C_ 81.9), C-5 (δ_C_ 47.8), and C-24 (δ_C_ 10.3), respectively, and the downfield shift (+15.9 ppm) exhibited by C-4 (δ_C_ 55.4) in comparison with the same carbon resonances in an oleanane skeleton bearing a Me-23 [15]. The α-configuration for the 23-CHO function was determined by the correlations of H-23 (δ_H_ 9.65) with H-3 (δ_H_ 4.04) and H-5 (δ_H_ 1.36) observed in the NOESY spectrum (Figure 2). The HMBC spectrum confirmed the 23-CHO function position by showing the correlations between H-23 (δ_H_ 9.65) and C-3 (δ_C_ 81.9), C-4 (δ_C_ 55.4) and C-24 (δ_C_ 10.3) (Figure 2). The assignments of the NMR signals of the aglycone moiety were derived from ^1^H-^1^H COSY, TOCSY, HSQC, HMBC, and NOESY spectra (Table 1). The aglycone of **1** was thus elucidated as gypsogenin [17,18,19]. The ^13^C-NMR shifts of C-3 at δ_C_ 81.9 and C-28 at δ_C_ 176.6 implied that sugar linkages were at both C-3 and C-28. The β-configuration for the 3-*O*-sugar moiety was deduced from the correlations of H-3 with H-23 and H-5 observed in the NOESY spectrum (Figure 2).

The monosaccharides of **1** were determined as L-arabinose (Ara), L-rhamnose (Rha), and D-glucose (Glc), in a ratio of 1:1:1 by acidic hydrolysis followed by gas chromatography (GC) analysis [20]. The ^1^H-NMR spectrum of compound **1** exhibited three anomeric protons at δ_H_ = 6.32 (d, *J* = 8.2 Hz), 6.17 (s) and 5.03 (d, *J* = 7.3 Hz), and one methyl group of 6-deoxy-hexopyranosyl moiety at δ_H_ 1.62 (d, *J* = 6.2 Hz). The α anomeric configuration of the Ara unit was deduced from the ^3^*J*_H-1/H-2_ (7.3 Hz) value observed in the ^4^*C*_1_ form. The Glc unit was determined to have a β anomeric configuration on the basis of its ^3^*J*_H-1/H-2_ coupling constant (8.2 Hz). Although the anomeric proton of the Rha moiety was observed as a singlet in the ^1^H-NMR spectrum, the ^13^C-NMR shift of Rha C-5 at δ_C_ = 69.6 indicated the α anomeric configuration [21,22]. The complete assignments of proton signals belonging to sugars were based on 2D NMR of ^1^H-^1^H COSY, TOCSY, and NOESY, and the carbon signals were assigned by HSQC and further confirmed by the HMBC spectrum (Table 2). The above NMR data indicated that all the monosaccharides were in their pyranose forms. The sequence and binding sites of the oligosaccharide chains were deduced from the HMBC spectrum (Figure 2). A cross peak between C-3 of the aglycone and H-1 of Ara revealed that Ara was connected to C-3 of the aglycone. Similarly, the linkage of Glc at C-28 of the aglycone was indicated by the cross peak Glc H-1/C-28, and the linkage of Rha at C-2 of Ara was indicated by the cross peak Rha H-1/Ara C-2. This conclusion was also supported by the NOESY correlations (Figure 2). On the basis of the above analysis, the structure of **1** was elucidated as 3β-*O*-α-L-rhamnopyranosyl-(1→2)-α-L-arabinopyranosyl gypsogenin 28-*O*-β-D-glucopyranosyl ester.

Saponin **2** was also obtained as a white powder. A pseudomolecular ion at *m*/*z* 1065.5252 (calcd. for 1065.5246 [M + Na]^+^) was found in HRESIMS, establishing the molecular formula of C_52_H_82_O_21_. By comparing with the 1D NMR data of **1**, the aglycone moiety of **2** was identical to **1** (Table 1), suggesting the same gypsogenin aglycone. The types of sugar units were determined as L-arabinose, D-xylose, L-rhamnose, and D-glucose in a 1:1:2:2 ratio by acid hydrolysis, followed by GC analysis. Six anomeric protons (δ_H_ 6.32 (s), 6.23 (d, *J* = 8.1 Hz), 5.85 (s), 5.35 (d, *J* = 7.7 Hz), 5.04 (d, *J* = 7.0 Hz), and 4.97 (d, *J* = 7.7 Hz)) and six anomeric carbons (δ_C_ 107.5, 104.9, 104.7, 102.6, 101.3, and 95.6) were observed in the NMR spectra of **2**. The linkage sites and the sequence of sugar moieties were deduced from the HMBC and NOESY correlations of signals at δ_H_ 5.04 (H-1 of Ara) with δ_C_ 81.8 (C-3 of the aglycone), δ_H_ 6.32 (H-1 of Rha I) with δ_C_ 75.5 (C-2 of Ara), δ_H_ 5.35 (H-1 of Xyl ) with δ_C_ 82.8 (C-3 of Rha I), δ_H_ 6.23 (H-1 of Glc I) with δ_C_ 176.7 (C-28 of the aglycone), δ_H_ 4.97 (H-1 of Glc II) with δ_C_ 69.1 (C-6 of Glc I), and δ_H_ 5.85 (H-1 of Rha II) with δ_C_ 78.1 (C-4 of Glc II) (Figure S1 in Supplementary data). Thus, the structure of **2** was assigned as 3β-*O*-β-D-xylopyranosyl-(1→3)-α-L-rhamnopyranosyl-(1→2)-α-L-arabinopyranosyl gypsogenin 28-*O*-α-L-rhamnopyranosyl-(1→4)-β-D-glucopyranosyl-(1→6)-β-D-glucopyranosyl ester.

Saponin **3** was obtained as a white powder. HRESIMS of **3** showed a quasi-molecular ion at *m*/*z* 1081.8564 (calcd. for 1081.8559 [M + Na]^+^), establishing the molecular formula of C_53_H_86_O_21_. The 1D NMR data of **3** exhibited signals for six tertiary methyl groups at 0.77 (H_3_-24), 0.92 (H_3_-25), 1.03 (H_3_-27), 1.10 (H_3_-26), 1.24 (H_3_-23), and 1.70 (H_3_-30), an exomethylene group at δ_H_ 4.70 and 4.85 with two olefinic carbon signals at δ_C_ 110.1 and 150.8, and one carbonyl signal at δ_C_ 174.9, which were characteristic of the Δ^20(29)^-lupane-type aglycone. The full assignments of the aglycone NMR signals were derived from 2D NMR data (Table 1), suggesting that the aglycone of **3** was betulinic acid [23,24]. The sugar moieties of **3** were determined as L-arabinose, L-rhamnose, and D-glucose in a ratio of 1:1:2 by acid hydrolysis, followed by GC analysis. Meanwhile, the 1D NMR spectra of **3** exhibited four anomeric protons at δ_H_ 6.34 (d, *J* = 8.2 Hz), 5.84 (s), 4.92 (d, *J* = 7.8 Hz), and 4.76 (d, *J* = 7.0 Hz), and four anomeric carbons at δ_C_ 107.5, 105.2, 102.7, and 95.3. The sequence and binding sites of the sugar units to each other and to the aglycone were deduced from the HMBC and NOESY spectra (Figure S2 in Supplementary data). On the basis of these findings, the structure of **3** was thus elucidated as 3β-*O*-α-L-arabinopyranosyl betulinic acid 28-*O*-α-L-rhamnopyranosyl-(1→4)-β-D-glucopyranosyl-(1→6)-β-D-glucopyranosyl ester. The lupane-type saponin was reported from this genus for the first time.

Saponin **4** was obtained as a white amorphous powder. In the positive-ion mode HRESIMS, a pseudomolecular ion peak at *m*/*z* 1097.5515 [M + Na]^+^ (calcd. for C_53_H_86_O_22_Na^+^, 1097.5508) was observed, suggesting a molecular formula C_53_H_86_O_22_. Seven tertiary methyl groups at δ_H_ 0.87 (H_3_-25), 0.99 (H_3_-30), 1.08 (H_3_-26), 1.13 (H_3_-29), 1.15 (H_3_-24), 1.29 (H_3_-23), and 1.31 (H_3_-27), one olefinic proton at δ_H_ 5.43 (1H, br s) with two typical olefinic carbon signals (at δ_C_ 122.6 and 144.1), and one carbonyl signal at δ_C_ 176.5 were observed in the 1D NMR spectra of **4**. This revealed **4** as an oleanane-type saponin. Due to the change of chemical shift of C-21 from δ_C_ 31.4 in oleanolic acid [25] to δ_C_ 73.2 (+41.8) and the other of carbons such as C-18 [δ_C_ 41.5 (−1.4)], C-19 [δ_C_ 41.3 (−5.0)], C-20 [δ_C_ 35.6 (+4.7)], C-22 [δ_C_ 39.5 (+6.3)], C-29 [δ_C_ 28.3 (−6.9)], and C-30 [δ_C_ 24.8 (+0.1)], C-21 must be an oxygen-bearing methylene carbon in the aglycone of **4**, which was confirmed by the HMBC experiment (Figure S3 in Supplementary data). The NOESY correlations between H-21 (δ_H_ 3.66) and H_3_-30 (δ_H_ 0.99) indicated the α-orientation of 21-OH (Figure S3 in Supplementary data). The assignments of the NMR signals associated with the aglycone moiety were derived from 2D NMR spectra (Table 1). These data revealed that the aglycone of **4** was 21α-hydroxy-oleanolic acid, which was in a good agreement when comparing the literature data [26,27]. Further comparison of the 1D NMR data assignable to the sugar part between **4** and **3** led to the determination of the same monosaccharide units and glycosylation sequence observed for both at C-3 and C-28 (Table 2). The conclusion was confirmed by the HMBC and NOESY spectra data (Figure S3 in Supplementary data). Therefore, the structure of saponin **4** was elucidated as 3β-*O*-α-L-arabinopyranosyl 21α-hydroxy-oleanolic acid 28-*O*-α-L-rhamnopyranosyl-(1→4)-β-D-glucopyranosyl-(1→6)-β-D-glucopyranosyl ester.

Saponin **5** was obtained as a white amorphous powder. The molecular formula of **5** was established as C_59_H_96_O_26_ from the quasi-molecular ion at *m*/*z* 1243.6094 (calcd. for 1243.6088 [M + Na]^+^) in HRESIMS. The 1D NMR spectra data assignable to the aglycone moiety of **5** were identical to those of **4** (Table 1), suggesting the same 21α-hydroxy-oleanolic acid aglycone. The spectra data assignable to the sugar moieties of **5** were similar to those of **4,** except for the presence of an additional α-L-rhamnopyranose moiety (Rha I). The downfield-shifted carbon signal of Ara C-2 (δ_C_ 75.5) in the ^13^C-NMR spectrum and the correlation between Rha I H-1 (δ_H_ 6.17) and Ara C-2 (δ_C_ 75.5) observed in the HMBC spectrum indicated that Rha I was attached to Ara C-2. The conclusion was supported by the NOESY spectrum (Figure S4 in Supplementary data). Thus, saponin **5** was elucidated as 3β-*O*-α-L-rhamnopyranosyl-(1→2)-α-L-arabinopyranosyl 21α-hydroxy-oleanolic acid 28-*O*-α-L-rhamnopyranosyl-(1→4)-β-D-glucopyranosyl-(1→6)-β-D-glucopyranosyl ester.

Additionally, the eight known saponins were identified as kalopanax saponin A (**6**) [28], pulsatilloside D (**7**) [29], 3β-*O*-{β-D-glucopyranosyl-(1→2)-*α*-L-arabinopyranosyl} oleanolic acid 28-*O*-β-D-glucopyranoside (**8**) [30], cauloside D (**9**) [31], cauloside F (**10**) [32], hederasaponin B (**11**) [33], 3β-*O*-{α-L-rhamnopyranosyl-(1→2)-[β-D-glucopyranosyl-(1→4)]-α-L-arabinopyranosyl} betulinic acid 28-*O*-α-L-rhamnopyranosyl-(1→4)-β-D-glucopyranosyl-(1→6)-β-D-glucopyranosyl ester (**12**) [34], and sieboldianoside A (**13**) [35]. This was accomplished by comparing their physicochemical and spectroscopic data with reported data [28,29,30,31,32,33,34,35].

For a long time, *A. rivularis* var. *flore-minore* was used as a folk medicine in Shaanxi Province for the treatment of hepatitis [9]. Inhibition of hepatic stellate cell (HSC) proliferation plays a key role in the pathogenesis of liver fibrosis caused by chronic hepatocellular damage [36]. HSC-T6, an immortalized rat hepatic stellate cell line, has generally been applied as a screening tool to evaluate the potential antifibrotic activity [37].

**Figure 3:**
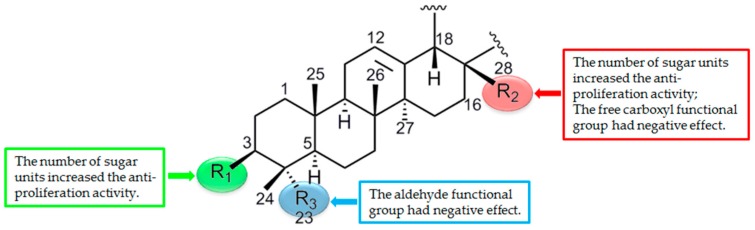
Brief structure–activity relationship analysis of the isolated saponins.

In this study, the anti-proliferative activity of isolated saponins on HSC-T6 cells was evaluated by 3-(4,5-dimethylthiazol-2-yl)-2,5-diphenyltetrazolium bromide MTT colorimetric assay. Colchicine was used as a positive control. As shown in Table 3, saponins **12** and **13** displayed potent anti-proliferative activity with IC_50_ values of 18.21 and 15.56 μM, respectively. Saponins **3**–**5** and **8**–**11** showed moderate antiproliferative activity with IC_50_ values ranging from 22.85 to 52.65 μM. These results revealed that the number of monosaccharides in sugar chains (both at C-3 and C-28) increased the anti-proliferative activity (Figure 3). The gypsogenin aglycone saponins (**1** and **2**) and monodesmosidic ones (**6** and **7**) were inactive, which suggested that the aldehyde functional group at C-23 and the free carboxyl functional group at C-28 had negative effects on anti-proliferative activity (Figure 3). It is worth mentioning that the monodesmosidic saponins (the sugar chain attached at C-3 and a free carboxylic acid at C-28) were cytotoxic against tumor cells in previous studies [10,14,15,17]. These opposing effects may be due to their different mechanisms against different types of cells. Nevertheless, the anti-proliferative activity of these saponins was very sensitive to their precise functionalization, especially for the sugar moieties. Further research is needed to approach a significative structure–activity relationship.

## 3. Experimental Section

### 3.1. General

Optical rotations were performed on a Perkin-Elmer 343 polarimeter (Perkin-Elmer Inc., Bridgeport, CT, USA). The ESIMS and HRESIMS were carried out on a Micromass Quattro mass spectrometer (Micromass Inc., Manchester, UK). NMR data were recorded on a Bruker AVANCE-500 spectrometer (Bruker Biospin AG, Fallanden, Switzerland). GC was tested on a Finnigan Voyager apparatus with an L-Chirasil-Val column (0.32 mm × 25 m; injector temperature: 230 °C; column temperature: 100–180 °C, rate 5 °C/min; column head pressure: 12 Pa; carrier gas: He, 2 mL/min) (Thermo Finnigan Inc, San Jose, CA, USA). Semi-preparative HPLC was performed on a Dionex P680 liquid chromatograph (Dionex Inc, Sunnyvale, CA, USA) equipped using a UV 170 UV/Vis detector at 206 nm. A YMC-Pack R&D ODS-A column (20 × 250 mm i.d., 5 μm, YMC Co., Ltd., Kyoto, Japan) was used. Materials for column chromatography (CC) were silica gel (10–40 μm, Qingdao Marine Chemical Inc., Qingdao, China), reversed phase silica gel ODS-A (50 μm, YMC Co., Ltd., Kyoto, Japan), and Sephadex LH-20 (40–70 μm, GE-Healthcare, Uppsala, Sweden). The Liebermann–Burchard reagent was prepared with acetic anhydride and sulfuric acid (Tianjin Fuyu Fine Chemical Co., Ltd., Tianjin, China), and the Molisch reagent was prepared with α-naphthol and sulfuric acid (Tianjin Fuyu Fine Chemical Co., Ltd., Tianjin, China).

### 3.2. Plant Material

The plant material was collected on Tsinling Mountains, Shaanxi Province, China, in October 2016. The plant was identified by Prof. Ji-Tao Wang at the Department of Pharmacognosy, School of Pharmacy, Shaanxi University of Chinese Medicine. A voucher specimen (No. 161018) was deposited in the Herbarium of Shaanxi University of Chinese Medicine.

### 3.3. Extraction and Isolation

The air-dried whole plants of *A. rivularis* var. *flore-minore* (5 kg) were powdered and extracted with 70% EtOH (3 × 8 L × 2 h) under reflux to give a crude extract (800 g), which was further suspended in water (8 L) and partitioned successively with petroleum ether (8 L × 2) and *n*-BuOH (8 L × 2). The *n*-BuOH extract (140 g) was divided into seven fractions (Fr. 1–Fr. 7) by using silica gel CC with a stepwise gradient of CHCl_3_–MeOH–H_2_O (10:1:0.04–6:4:0.7). Fr. 4 (16.5 g) was separated by silica gel CC with a CHCl_3_–MeOH–H_2_O gradient (10:1:0.1–7:3:0.4) to give six sub-fractions (Fr. 4.1–Fr. 4.6) and further purified by on a Sephadex LH-20 column in MeOH. Saponins **6** (37 mg, *t*_R_ 17.4 min) and **7** (26 mg, *t*_R_ 23.5 min) were obtained from Fr. 4.3 (2.2 g) by semi-preparative HPLC (MeOH–H_2_O (82:18), 7.2 mL/min). Fr. 4.4 (3.2 g) and Fr. 4.5 (2.5 g) were subjected to semi-preparative HPLC to give saponin **8** (18 mg, MeOH–H_2_O (61:39), 8 mL/min, *t*_R_ 19.2 min from Fr. 4.4), saponin **1** (22 mg, MeOH–H_2_O (60:40), 8.0 mL/min, *t*_R_ 20.5 min from Fr. 4.5) and saponin **9** (20 mg, MeOH–H_2_O (60:40), 8.0 mL/min, *t*_R_ 24.5 min from Fr. 4.5). Fr. 5 (23 g) was separated by silica gel CC with a stepwise gradient of CHCl_3_–MeOH–H_2_O gradient (10:1:0.1–7:3:0.4) to yield eight sub-fractions (Fr. 5.1–Fr. 5.8). Fr. 5.2 (1.8 g) and Fr. 5.3 (2.4 g) were purified by gel CC over Sephadex LH-20 (MeOH), and further submitted to semi-preparative HPLC to give saponin **2** (24 mg, MeOH–H_2_O (72:28), 8 mL/min, *t*_R_ 19.0 min) and **3** (18 mg, MeOH–H_2_O (76:24), 8 mL/min, *t*_R_ 17.5 min), respectively. Saponins **10** (25 mg, *t*_R_ 18.6 min) and **4** (16 mg, *t*_R_ 26.9 min) were obtained from Fr. 5.4 by semi-preparative HPLC (MeOH–H_2_O (58:42), 7.5 mL/min). Fr. 5.5 (4.3 g) was subjected to ODS CC with a MeOH–H_2_O (1:10–3:1) gradient to afford Fr. 5.5.1–Fr. 5.5.4. Saponins **11** (35 mg, *t*_R_ 23.5 min) and **5** (20 mg, *t*_R_ 27.3 min) were obtained from Fr. 5.5.3 by semi-preparative HPLC (MeOH–H_2_O (58:42), 8 mL/min). Fr. 6 (16 g) was separated by silica gel CC with a stepwise gradient of CHCl_3_–MeOH–H_2_O (8:2:0.2–6:4:0.5) to yield Fr. 6.1–Fr. 6.5. Fr. 6.2 (4.5 g) was subjected to ODS CC with a stepwise MeOH–H_2_O (1:4–4:1) gradient to afford four Fr. 6.2.1–Fr. 6.2.4. Saponins **12** (18 mg, MeOH–H_2_O (70:30), 8 mL/min, *t*_R_ 15.2 min) and **13** (26 mg, MeOH–H_2_O (68:32), 8 mL/min, *t*_R_ 18.5 min) were obtained by semi-preparative HPLC from Fr. 6.2.2 and Fr. 6.2.3, respectively. The purity of all compounds was assessed by HPLC as more than 95%.

### 3.4. Compound Characterization Data

Compound **1**: White amorphous powder; [α${]}_{D}^{22}$ +21.5 (*c* 0.18, MeOH); for ^1^H- and ^13^C-NMR spectroscopic data, see Table 1 and Table 2; key HMBC and NOESY correlations, see Figure 2; HRESIMS (pos. ion mode) *m*/*z* 933.4829 [M + Na]^+^ (calcd. for C_47_H_74_NaO_17_, 933.4824); ESIMS (pos. ion mode) *m*/*z* 933 [M + Na]^+^.

Compound **2**: White amorphous powder; [α${]}_{D}^{22}$ −11.6 (*c* 0.15, MeOH); for ^1^H- and ^13^C-NMR spectroscopic data, see Table 1 and Table 2; key HMBC and NOESY correlations, see Figure S1 in Supplementary data; HRESIMS (pos. ion mode) *m*/*z* 1065.5252 [M + Na]^+^ (calcd. for C_52_H_82_NaO_21_, 1065.5246); ESIMS (pos. ion mode) *m*/*z*1065 [M + Na]^+^.

Compound **3**: White amorphous powder; [α${]}_{D}^{22}$ +17.2 (*c* 0.20, MeOH); for ^1^H- and ^13^C-NMR spectroscopic data, see Table 1 and Table 2; key HMBC and NOESY correlations, see Figure S2 in Supplementary data; HRESIMS (pos. ion mode) *m*/*z* 1081.8564 [M + Na]^+^ (calcd. for C_53_H_86_NaO_21_, 1081.8559); ESIMS (pos. ion mode) *m*/*z*1081 [M + Na]^+^.

Compound **4**: White amorphous powder; [α${]}_{D}^{22}$ +12.3 (*c* 0.14, MeOH); for ^1^H- and ^13^C-NMR spectroscopic data, see Table 1 and Table 2; key HMBC and NOESY correlations, see Figure S3 in Supplementary data; HRESIMS (pos. ion mode) *m*/*z* 1097.5515 [M + Na]^+^ (calcd. for C_53_H_86_NaO_22_, 1097.5508); ESIMS (pos. ion mode) *m*/*z* 1097 [M + Na]^+^.

Compound **5**: White amorphous powder; [α${]}_{D}^{22}$ −11.4 (*c* 0.15, MeOH); for ^1^H- and ^13^C-NMR spectroscopic data, see Table 1 and Table 2; key HMBC and NOESY correlations, see Figure S4 in Supplementary data; HRESIMS (pos. ion mode) *m*/*z* 1243.6094 [M + Na]^+^ (calcd. for C_59_H_96_NaO_26_, 1243.6088); ESIMS (pos. ion mode) *m*/*z* 1243 [M + Na]^+^.

### 3.5. Acid Hydrolysis and GC Analysis of the Sugar Moieties in ***1**–**5***

Saponins **1**–**5** (each 4 mg) were hydrolyzed with 2 mol/L CF_3_COOH (5 mL) at 100 °C for 3 h, respectively. The mixture of reactants was evaporated in vacuo, and the residue was partitioned between H_2_O and CHCl_3_ three times. The residue was dissolved in pyridine (4 mL) and 1-(trimethylsilyl)-imidazole (0.5 mL). The reaction mixture was stirred at 60 °C for 5 min and dried with a stream of N_2_. Then, the residue was partitioned between H_2_O and hexane, and the latter layer was subjected to GC analysis with an l-Chirasil-Val column. The configurations of the monosaccharide units were established by comparing retention times with those of the trimethylsilylated derivatives prepared in the same manner from the authentic standard monosaccharides [38]. Retention times for authentic samples were detected at 8.92 and 9.95 min (d-arabinose), 9.60 and 10.38 min (l-rhamnose), 10.91 and 12.15 min (d-xylose), and 14.82 min (d-glucose), respectively. l-arabinose, l-rhamnose, and d-glucose were measured in a ratio of 1:1:1 for **1**, 1:1:2 for **3** and **4**, and 1:2:2 for **5**, while the sugar moieties of **2** were identified as l-arabinose, l-rhamnose d-xylose and d-glucose in the ratio of 1:1:1:1.

### 3.6. HSC-T6 Cell Culture and Cell Viability Assay

The anti-proliferative activity of saponins **1**–**13** was evaluated on hepatic stellate cell (HSC)-T6 cells (Chinese Academy of Science Committee Type Culture Collection Cell Bank, Shanghai, China). The HSC-T6 cells were found to be mycoplasma free by PCR. HSC-T6 cells were maintained in dulbecco’s modified Eagle’s medium (DMEM) (Sigma-Aldrich, St. Louis, MO, USA), which was supplemented with 10% heat-inactivated fetal bovine serum, 100 IU/mL penicillin (Sigma-Aldrich), and 100 μg/mL streptomycin (Sigma-Aldrich) at 37 °C in a humidified atmosphere of 95% air–5% CO_2_. Cell viability was evaluated by MTT colorimetric assay, with colchicine (Sigma-Aldrich) used as a positive control. The cells were seeded in 96-well plates at a density of 5 × 10^4^ cells/mL and incubated for 24 h. Each saponin was dissolved in DMSO and diluted with distilled water to reach the desired concentrations. The cells were treated with these drugs (0.5, 1, 5, 10, 20, 40, and 80 μM) in triplex wells for 48 h at 37 °C in a humidified 5% CO_2_ atmosphere. An amount of 20 μL MTT (Sigma-Aldrich) reagent solved in PBS was added to each well (final concentration = 5 mg/mL), and further incubated for 4 h. After removing the supernatant, DMSO was added to solubilize the formazan crystals. The optical density of each well was measured with a Bio-Rad 680 microplate reader at 560 nm. Anti-proliferative activity was expressed as the concentration of compound producing 50% of cell inhibitory rate (IC_50_).

## 4. Conclusions 

In this study, thirteen triterpenoid saponins, including five new ones, were isolated from *A. rivularis* var. *flore-minore*. All the structures were established on the basis of extensive spectroscopic studies along with MS analyses and acid hydrolysis. Five kinds of aglycones were identified, i.e., gypsogenin, betulinic acid, 21-hydroxy-oleanolic acid, hederagenin, and oleanolic acid. The lupane-type saponins (**3** and **12**) were reported from the *Anemone* genus for the first time. The anti-proliferative activity of all isolated saponins was evaluated on hepatic stellate cells (HSC-T6). The preliminary structure–activity relationship analyses revealed that the more monosaccharides the saponins possessed, the stronger the anti-proliferative activity exhibited. This work will not only enrich the diversity of triterpenoid saponins of this genus, but will also provide a reference for the discovery of potential lead compounds for liver disease drug development.

## Figures and Tables

**Table 1:** ^1^H- (500 MHz) and ^13^C-NMR (125 MHz) data for the aglycone moieties of **1**–**5** in pyridine-*d*_5_.

C	1		2		3		4		5	
	δ_C_	δ_H_	δ_C_	δ_H_	δ_C_	δ_H_	δ_C_	δ_H_	δ_C_	δ_H_
1	38.5	0.97, 1.55 m	38.6	1.00, 1.59 m	39.4	1.60, 0.89 m	38.8	0.93, 1.45 m	38.9	0.95, 1.46 m
2	25.7	1.84, 2.09 m	25.6	1.87, 2.10 m	26.8	2.18, 1.86 m	26.6	1.84, 2.06 m	26.7	1.87, 2.08 m
3	81.9	4.04 m	81.8	4.06 m	88.7	3.34 m	88.7	3.26 dd (3.7, 11.6)	88.8	3.28 dd (3.9, 11.6)
4	55.4	-	55.6	-	39.6	-	39.4	-	39.5	-
5	47.8	1.36 m	48.0	1.37 m	56.0	0.74 m	56.0	0.77 d (11.5)	56.1	0.79 d (11.6)
6	20.6	0.98, 1.39 m	20.7	0.99, 1.42 m	18.4	1.67, 1.45 m	18.4	1.25, 1.44 m	18.5	1.26, 1.46 m
7	32.6	1.17, 1.42 m	32.7	1.19, 1.42 m	34.5	1.30, 1.28 m	33.1	1.25, 1.40 m	33.2	1.25, 1.42 m
8	40.0	-	40.2	-	41.1	-	39.7	-	39.9	-
9	47.8	1.69 m	48.1	1.70 m	50.8	1.30 m	48.1	1.66 m	48.2	1.68 m
10	36.0	-	36.2	-	37.1	-	36.9	-	37.0	-
11	23.2	1.90, 1.99 m	23.4	1.91, 2.03 m	21.1	1.31, 1.13 m	23.7	1.86, 1.92 m	23.8	1.87, 1.94 m
12	122.7	5.41 br s	122.6	5.42 br s	26.0	1.84, 1.13 m	122.6	5.43 br s	122.8	5.44 br s
13	144.1	-	144.2	-	38.3	2.64 m	144.1	-	144.3	-
14	41.6	-	41.8	-	42.7	-	42.2	-	42.4	-
15	28.1	1.13, 2.04 m	28.3	1.15, 2.06 m	30.1	2.00, 1.18 m	28.5	1.15, 2.30 m	28.6	1.16, 2.32 m
16	23.2	1.77, 2.01 m	23.3	1.78, 2.05 m	32.4	2.63, 1.48 m	27.0	2.35, 3.08 m	27.1	2.37, 3.11 m
17	47.2	-	47.3	-	57.0	-	47.1	-	47.2	-
18	41.5	3.14 dd (3.3, 13.4)	41.7	3.15 dd (3.9, 13.5)	49.8	1.71 m	41.5	3.36 dd (3.3, 14.0)	41.7	3.38 dd (3.4, 13.9)
19	46.1	1.23, 1.74 m	46.2	1.24, 1.75 m	47.4	3.36 m	41.3	1.70, 1.21 m	41.5	1.73, 1.22 m
20	30.6	-	30.7	-	150.8	-	35.6	-	35.7	-
21	33.8	1.12, 1.34 m	34.0	1.13, 1.36 m	30.8	2.15, 1.40 m	73.2	3.66 br s	73.4	3.67 br s
22	32.3	1.71, 1.89 m	32.4	1.73, 1.91 m	37.1	2.19, 1.45 m	39.5	2.25, 2.27 m	39.6	2.26, 2.28 m
23	206.5	9.65 s	206.6	9.66 s	28.1	1.24 s	28.1	1.29 s	28.2	1.30 s
24	10.3	1.34 s	10.5	1.35 s	16.3	0.77 s	17.2	1.15 s	17.3	1.16 s
25	15.6	0.89 s	15.7	0.89 s	16.7	0.92 s	15.5	0.87 s	15.7	0.88 s
26	17.3	1.05 s	17.5	1.06 s	16.4	1.10 s	17.4	1.08 s	17.6	1.10 s
27	26.2	1.19 s	26.3	1.22 s	14.8	1.03 s	25.5	1.31 s	25.7	1.33 s
28	176.6	-	176.7	-	174.9	-	176.4	-	176.6	-
29	33.0	0.86 s	33.1	0.87 s	110.1	4.85, 4.70(br s)	28.3	1.13 s	28.5	1.14 s
30	23.7	0.87 s	23.8	0.88 s	19.4	1.70 s	24.8	0.99 s	25.1	1.01 s

**Table 2:** ^1^H- (500 MHz) and ^13^C-NMR (125 MHz) data for the sugar moieties of **1**–**5** in pyridine-*d*_5_.

C	1		2		3		4		5	
	δ_C_	δ_H_	δ_C_	δ_H_	δ_C_	δ_H_	δ_C_	δ_H_	δ_C_	δ_H_
3-*O*-sugar										
Ara										
1	104.6	5.03 d (7.3)	104.7	5.04 d (7.0)	107.5	4.76 d (7.0)	107.4	4.77 d (6.8)	104.5	5.03 d (7.3)
2	75.7	4.43 m	75.5	4.53 m	72.9	4.40 m	72.8	4.41 m	75.5	4.43 m
3	74.7	4.16 m	75.2	4.04 m	74.6	4.13 m	74.4	4.14 m	74.6	4.16 m
4	69.3	4.20 m	69.9	4.12 m	69.5	4.29 m	69.5	4.27 m	69.3	4.20 m
5	65.7	3.75, 4.42 m	66.3	3.56, 4.32 m	67.1	3.80, 4.28 m	67.0	3.81, 4.26 m	65.5	3.76, 4.42 m
Rha I										
1	101.6	6.17 s	101.3	6.32 s					101.7	6.17 s
2	72.3	4.71 br s	71.9	4.89 brs					72.3	4.71 br s
3	72.5	4.59 m	82.8	4.75 m					72.4	4.59 m
4	74.1	4.28 m	72.9	4.45 m					74.0	4.27 m
5	69.6	4.61 m	69.6	4.62 m					69.8	4.61 m
6	18.5	1.62 d (6.2)	18.4	1.52 d (6.2)					18.6	1.62 d (6.2)
Xyl										
1			107.5	5.35 d (7.7)						
2			75.6	4.05 m						
3			78.4	4.15 m						
4			71.1	4.19 m						
5			67.4	3.69, 4.30 m						
28-*O*-sugar										
Glc I										
1	95.7	6.32 d (8.2)	95.6	6.23 d (8.1)	95.3	6.34 d (8.2)	95.5	6.24 d (8.2)	95.6	6.23 d (8.1)
2	74.1	4.19 m	73.8	4.08 m	74.0	4.08 m	73.7	4.07 m	73.8	4.10 m
3	79.2	4.02 m	78.7	4.15 m	78.7	4.20 m	78.6	4.16 m	78.7	4.17 m
4	71.1	4.34 m	70.8	4.28 m	70.9	4.29 m	70.8	4.27 m	70.7	4.30 m
5	78.9	4.26 m	78.0	4.08 m	78.0	4.08 m	77.9	4.06 m	78.0	4.09 m
6	62.3	4.40, 4.43 m	69.1	4.31, 4.63 m	69.4	4.30, 4.66 m	69.1	4.27, 4.64 m	69.0	4.30, 4.63 m
Glc II										
1			104.9	4.97 d (7.7)	105.2	4.92 d (7.8)	104.8	4.97 d (7.8)	104.9	4.98 d (7.8)
2			75.3	3.92 m	75.3	3.92 m	75.2	3.91 m	75.3	3.92 m
3			76.5	4.12 m	76.4	4.11 m	76.4	4.12 m	76.5	4.14 m
4			78.1	4.40 m	78.2	4.39 m	78.1	4.37 m	78.2	4.39 m
5			77.1	3.62 m	77.2	3.63 m	77.0	3.64 m	77.1	3.64 m
6			61.2	4.07, 4.18 m	61.3	4.06, 4.18 m	61.2	4.04, 4.19 m	61.3	4.06, 4.20 m
Rha II										
1			102.6	5.85 s	102.7	5.84 s	102.6	5.84 s	102.7	5.86 s
2			72.6	4.66 m	72.6	4.65 m	72.5	4.63 m	72.6	4.64 m
3			72.7	4.53 m	72.8	4.50 m	72.7	4.50 m	72.7	4.54 m
4			74.1	4.31 m	74.0	4.30 m	74.0	4.30 m	74.1	4.32 m
5			70.3	4.94 m	70.3	4.95 m	70.3	4.92 m	70.4	4.95 m
6			18.5	1.68 d (6.2)	18.5	1.69 m	18.5	1.67 d (6.2)	18.6	1.66 d (6.2)

**Table 3:** Anti-proliferation activity of saponins **1**–**13** on HSC-T6 cells (mean ± SD, *n* = 3).

Saponins ^a^	IC_50_ (μM)	Saponins ^a^	IC_50_ (μM)
3	28.62 ± 0.76	10	38.62 ± 1.58
4	22.85 ± 2.21	11	25.43 ± 2.86
5	25.74 ± 1.34	12	18.21 ± 0.92
8	52.65 ± 3.19	13	15.56 ± 1.58
9	43.65 ± 2.85	Colchicine ^b^	9.35 ± 0.25

^a^ Compounds **1**, **2**, **6**, and **7** were inactive (IC_50_ >80 μM); ^b^ Colchicine was used as a positive control.

## Supplementary Materials

Supplementary data associated with this article can be found online. Table S1. ^13^C-NMR (125 MHz) chemical shifts of compounds **6**–**9** in pyridine-*d*_5_; Table S2. 13C-NMR (125 MHz) chemical shifts of compounds **10**–**13** in pyridine-*d*_5_; Figure S1. Key NOESY and HMBC correlations for compound **2**; Figure S2. Key NOESY and HMBC correlations for compound **3**; Figure S3. Key NOESY and HMBC correlations for compound **4**; Figure S4. Key NOESY and HMBC correlations for compound **5**.
