# Supplementary material for: Triterpenoid Saponins from Anemone rivularis var. Flore-Minore and Their Anti-Proliferative Activity on HSC-T6 Cells
Source: Molecules. 2018 Feb 23;23(2):491. doi: 10.3390/molecules23020491 (PMC6017197; doi:10.3390/molecules23020491)
Supplement: Supplementary file 1 [file molecules-23-00491-s001.docx]

**Supporting Information**

**Triterpenoid saponins from *Anemone rivularis* var*. flore-minore* and their anti-proliferative activity on HSC-T6 Cells**

**Xiao-Yang Wang ^1, †^, Hui Gao ^2, †^, Xiao-Jie Xie ^1^, Jirimubatu Jurhiin ^3^, Mu-Zi-He Zhang ^1^, Yan-Ping Zhou ^1^, Rui Liu^1^,** **Meng Ning, Jin Han ^1,^*, Hai-Feng Tang ^4,^***

^1^ Department of Pharmacy, People's Liberation Army Institute of Chinese Medicine, Beijing 100039, China; steveplum@sina.com (X.-Y.W.); xiexiaojie1991@163.com (X.-J.X.); zhangmuzihe@163.com (M.-Z.-H.Z.); zhouyp0102@126.com (Y.-P.Z.); liurui198003@yeah.net; lemon1572@163.com (M.N.)

^2^ People's Liberation Army Institute of Organ Transplantation, Beijing 100091, China; gaohui309@163.com

^3^ Academy of Mongolian Medicine, Inner Mongolia Medical University, Hohhot 010110, China; jormbut@163.com

^4^ Institute of Materia Medica, School of Pharmacy, The Fourth Military Medical University, Xi’an 710032, China

***** Correspondence: hanjin302pharm@126.com (J.H.); tanghf71@fmmu.edu.cn (H.-F.T.); Tel.: +86-10-6693-3225 (J.H.); +86-29-8477-4748 (H.-F.T.)

† The authors contribute equally to this work.

**Contents**

**Identification data of compounds 6**−**13** 3

**Table S1** ^13^C-NMR (125 MHz) chemical shifts of compounds **6**−**9** in pyridine-*d*_5_ 5

**Table S2** ^13^C-NMR (125 MHz) chemical shifts of compounds **10**−**13** in pyridine-*d*_5_ 6

**Figure** **S1** Key NOESY and HMBC correlations for compound **2**. 7

**Figure** **S2** Key NOESY and HMBC correlations for compound **3**. 7

**Figure** **S3** Key NOESY and HMBC correlations for compound **4**. 8

**Figure** **S4** Key NOESY and HMBC correlations for compound **5**. 8

Compound **6**: kalopanaxsaponin A

White amorphous powder; [*α*] +19.6 (*c* 0.15, MeOH); ESI-MS (pos. ion mode) *m/z* 773 [M+Na]^+^; ESI-MS (neg. ion mode) *m/z* 749 [M−H]^−^; ^1^H-NMR (500 MHz, pyridine-*d*_5_) *δ*: 0.89, 0.91, 0.96, 0.99, 1.03, 1.20 (each 3H, s, CH_3_), 1.62 (3H, d, *J* = 6.2 Hz, CH_3_ of Rha), 3.26 (1H, dd, *J* = 4.0, 13.8 Hz, H-3), 5.09 (1H, d, *J* = 6.3 Hz, H-1 of Ara), 5.43(1H, br s, H-12), 6.23 (1H, s, H-1 of Rha); ^13^C-NMR data, see Table S1.

Compound **7**: pulsatilloside D

White amorphous powder; [*α*] +11.2 (*c* 0.15, MeOH); ESI-MS (pos. ion mode) *m/z* 789 [M+Na]^+^; ESI-MS (neg. ion mode) *m/z* 765 [M−H]^−^; ^1^H-NMR (500 MHz, pyridine-*d*_5_) *δ*: 0.94, 0.94, 1.00, 1.03, 1.04, 1.22 (each 3H, s, CH_3_), 3.29 (1H, dd, *J* = 3.9, 13.8 Hz,H-18), 5.04 (1H, d, *J* = 6.7 Hz, H-1 of Ara), 5.22 (1H, d, *J* = 7.7 Hz, H-1 of Glc), 5.45(1H, br s, H-12); ^13^C-NMR data, see Table S1.

Compound **8**：3*β*-O-{*β*-D-glucopyranosyl-(1→2)-*α*-L-arabinopyranosyl} oleanolic acid 28-O-*β*-D- glucopyranoside

White amorphous powder; [*α*]−10.1 (*c* 0.25, MeOH); ESI-MS (pos. ion mode) *m/z* 935 [M+Na]^+^; ESI-MS (neg. ion mode) *m/z* 911 [M−H]^−^; ^1^H-NMR (500 MHz, pyridine-*d*_5_) *δ*: 0.84, 0.87, 0.89, 1.01, 1.08, 1.19, 1.22 (each 3H, s, CH_3_), 3.18 (1H, m ,H-18), 4.93 (1H, d, *J* = 5.7 Hz, H-1 of Ara), 5.16 (1H, d, *J* = 7.7 Hz, H-1 of 3-O-Glc), 5.41 (1H, br s, H-12), 6.31 (1H, *J* = 8.1 Hz, H-1 of 28-O-Glc); for ^13^C-NMR spectroscopic data, see Table S1.

Compound **9**：cauloside D

White amorphous powder; [*α*] +13.6 (*c* 0.20, MeOH); ESI-MS (pos. ion mode) *m/z* 1097 [M+Na]^+^; ESI-MS (neg. ion mode) *m/z* 1073 [M−H]^−^; ^1^H-NMR (500 MHz, pyridine-*d_5_*) *δ*: 0.85, 0.85, 0.90, 0.95, 1.09, 1.13(each 3H, s, CH_3_), 1.68(3H, d, *J* = 6.1 Hz, CH_3_ of Rha), 3.13 (1H, dd, *J* = 3.3, 13.3 Hz, H-18), 4.91 (1H, d, *J* = 7.0 Hz, H-1 of Ara), 4.92 (1H, d, *J* = 7.8 Hz, H-1 of Glc II), 5.37 (1H, br s, H-12), 5.81 (1H, s, H-1 of Rha), 6.21 (1H, d, *J* = 8.1 Hz, H-1 of Glc I); for ^13^C-NMR spectroscopic data, see Table S1.

Compound **10**：cauloside F

White amorphous powder; [*α*]+10.8 (*c* 0.20, MeOH); ESI-MS (pos. ion mode) *m/z* 1237 [M+Na]^+^; ESI-MS (neg. ion mode) *m/z* 1235 [M−H]^−^; ^1^H-NMR (500 MHz, pyridine-*d_5_*) *δ*: 0.85, 0.86, 0.95, 1.01, 1.10, 1.34(each 3H, s, CH_3_), 1.69 (3H, d, *J* = 6.1 Hz, CH_3_ of Rha), 3.15 (1H, dd, *J* = 3.8, 13.7 Hz, H-18), 4.90 (1H, d, *J* = 7.0 Hz, H-1 of Ara), 4.95 (1H, d, *J* = 7.8 Hz, H-1 of Glc II), 5.15 (1H, d, *J* = 7.7 Hz, H-1 of Glc I), 5.38 (1H, br s, H-12), 5.81 (1H, s, H-1 of Rha), 6.21 (1H, d, *J* = 8.0 Hz, H-1 of Glc II); for ^13^C-NMR spectroscopic data, see Table S2.

Compound **11**：hederasaponin B

White amorphous powder; [*α*]−15.5 (*c* 0.25, MeOH); ESI-MS (pos. ion mode) *m/z* 1227 [M+Na]^+^; ESI-MS (neg. ion mode) *m/z* 1203 [M−H]^−^; ^1^H-NMR (500 MHz, pyridine-*d*_5_) *δ*: 0.85, 0.87, 0.89, 1.03, 1.08, 1.19, 1.22 (each 3H, s, CH_3_), 1.67 (3H, d, *J* = 6.2 Hz, CH_3_ of Rha I), 1.68 (3H, d, *J* = 6.1 Hz, CH_3_ of Rha II), 3.15 (1H, m, H-18), 4.92 (1H, d, *J* = 6.1 Hz, H-1 of Ara), 4.94 (1H, d, *J* = 7.8 Hz, H-1 of Glc III), 5.41 (1H, br s, H-12), 5.82 (1H, s, H-1 of Rha II), 6.22 (1H, d, *J* = 8.1 Hz, H-1 of Glc II), 6.31 (1H, br s, H-1 of Rha I); for ^13^C-NMR spectroscopic data, see Table S2.

Compound **12**：3*β*-*O*-{α-L-rhamnopyranosyl-(1→2)-[β-D-glucopyranosyl-(1→4)]-α-L-arabinopyranosyl} betulinic acid 28-*O*-α-L-rhamnopyranosyl-(1→4)-β-D-glucopyranosyl-(1→6)-β-D-glucopyranosyl ester

White amorphous powder; [*α*]−18.5 (*c* 0.22, MeOH); ESI-MS (pos. ion mode) *m/z* 1389 [M+Na]^+^; ESI-MS (neg. ion mode) *m/z* 1365 [M−H]^−^; ^1^H-NMR (500 MHz, pyridine-*d_5_*) *δ*: 0.77, 0.93, 1.03, 1.11, 1.23, 1.70 (each 3H, s, CH_3_), 1.62 (3H, d, *J*= 6.2 Hz, CH_3_ of 3-*O*-Rha), 1.68 (3H, d, *J*= 6.2 Hz, CH_3_ of 28-*O*-Rha), 3.36 (1H, m, H-19), 4.70, 4.85 (each H, br s, H_2_-29), 4.76 (1H, d, *J*= 6.2 Hz, H-1 of Ara), 4.92 (1H, d, *J*= 7.8 Hz, H-1 of 28-*O*-Glc III), 5.13 (1H, d, *J*= 7.9 Hz, H-1 of 3-*O*-Glc I), 5.83 (1H, s, H-1 of 28-*O*- Rha), 6.15 (1H, s, H-1 of 3-*O*-Rha), 6.33 (1H, d, *J*= 8.2 Hz, H-1 of 28-*O*-Glc II); for ^13^C-NMR spectroscopic data, see Table S2.

Compound **13**：sieboldianoside A

White amorphous powder; [*α*] −19.5 (*c* 0.37, MeOH); ESI-MS (pos. ion mode) *m/z* 1375 [M+Na]^+^; ^1^H-NMR (500 MHz, pyridine-*d_5_*) *δ*: 0.84, 0.85, 0.95, 1.10, 1.13, 1.17 (each 3H, s, CH_3_), 1.54 (3H, d, *J* = 6.2 Hz, CH_3_ of Rha I), 1.68 (3H, d, *J* = 6.2 Hz, CH_3_ of Rha II), 3.14 (1H, dd, *J* = 3.7, 13.7Hz, H-18), H-1 of Ara (overlapped), H-1 of Glc III (overlapped), 5.34 (1H, d, *J* = 7.9 Hz, H-1 of Xyl), 5.35 (1H, br s, H-12), 5.84 (1H, s, H-1 of Rha II), 6.23 (1H, d, *J* = 8.2 Hz, H-1 of 28-O-Glc II), 6.32 (1H, s, H-1 of Rha I); for ^13^C-NMR spectroscopic data, see Table S2.

**Table S1** ^13^C-NMR (125 MHz) chemical shifts of saponins **6**−**9** in pyridine-*d*_5_

| C | **6** | | **7** | **8** | **9** |  | C | **6** | **7** | **8** | **9** |
| --- | --- | --- | --- | --- | --- | --- | --- | --- | --- | --- | --- |
| 1 | 38.9 | | 38.7 | 38.7 | 38.8 |  | Rha I |  |  |  |  |
| 2 | 26.3 | | 26.2 | 26.4 | 26.0 |  | 1 | 101.6 |  |  |  |
| 3 | 81.1 | | 81.0 | 88.8 | 81.9 |  | 2 | 72.2 |  |  |  |
| 4 | 43.5 | | 43.4 | 39.4 | 43.5 |  | 3 | 72.5 |  |  |  |
| 5 | 47.7 | | 47.7 | 55.8 | 47.6 |  | 4 | 74.0 |  |  |  |
| 6 | 18.1 | | 18.1 | 18.5 | 18.1 |  | 5 | 69.6 |  |  |  |
| 7 | 32.8 | | 32.8 | 33.1 | 32.8 |  | 6 | 18.6 |  |  |  |
| 8 | 39.7 | | 39.7 | 39.8 | 39.9 |  | Glc I |  |  |  |  |
| 9 | 48.1 | | 48.1 | 48.0 | 48.2 |  | 1 |  | 106.0 | 106.0 |  |
| 10 | 36.8 | | 36.9 | 36.9 | 36.9 |  | 2 |  | 76.3 | 76.4 |  |
| 11 | 23.8 | | 23.8 | 23.7 | 23.8 |  | 3 |  | 78.3 | 78.2 |  |
| 12 | 122.5 | | 122.5 | 122.8 | 122.9 |  | 4 |  | 71.4 | 71.5 |  |
| 13 | 144.8 | | 144.8 | 144.1 | 144.1 |  | 5 |  | 78.2 | 78.1 |  |
| 14 | 42.1 | | 42.1 | 42.0 | 42.1 |  | 6 |  | 62.5 | 62.5 |  |
| 15 | 28.3 | | 28.3 | 28.1 | 28.3 |  | 28-*O*-sugar |  |  |  |  |
| 16 | 23.6 | | 23.6 | 23.3 | 23.3 |  | Glc II |  |  |  |  |
| 17 | 46.6 | | 46.6 | 47.0 | 47.0 |  | 1 |  |  | 95.7 | 95.6 |
| 18 | 41.9 | | 41.9 | 41.6 | 41.6 |  | 2 |  |  | 74.2 | 73.9 |
| 19 | 46.4 | | 46.3 | 46.2 | 46.1 |  | 3 |  |  | 79.0 | 78.7 |
| 20 | 30.9 | | 30.9 | 30.7 | 30.7 |  | 4 |  |  | 71.1 | 70.8 |
| 21 | 34.0 | | 34.2 | 33.9 | 33.9 |  | 5 |  |  | 79.4 | 78.0 |
| 22 | 33.2 | | 33.2 | 32.5 | 32.5 |  | 6 |  |  | 62.2 | 69.1 |
| 23 | 63.9 | | 63.9 | 28.1 | 64.3 |  | Glc III |  |  |  |  |
| 24 | 13.9 | | 14.0 | 16.7 | 13.6 |  | 1 |  |  |  | 104.8 |
| 25 | 16.0 | | 16.0 | 15.5 | 16.2 |  | 2 |  |  |  | 75.3 |
| 26 | 17.4 | | 17.4 | 17.4 | 17.5 |  | 3 |  |  |  | 76.5 |
| 27 | 26.1 | | 26.1 | 26.0 | 26.0 |  | 4 |  |  |  | 78.2 |
| 28 | 180.3 | | 180.2 | 176.4 | 176.4 |  | 5 |  |  |  | 77.1 |
| 29 | 33.2 | | 33.2 | 33.1 | 33.1 |  | 6 |  |  |  | 61.2 |
| 30 | 23.7 | | 23.7 | 23.6 | 23.6 |  | Rha II |  |  |  |  |
| 3-*O*-sugar | | |  |  |  |  | 1 |  |  |  | 102.7 |
| Ara | |  |  |  |  |  | 2 |  |  |  | 72.5 |
| 1 | | 104.4 | 104.0 | 104.8 | 106.6 |  | 3 |  |  |  | 72.7 |
| 2 | | 75.6 | 81.4 | 81.0 | 73.1 |  | 4 |  |  |  | 74.0 |
| 3 | | 74.7 | 73.7 | 73.4 | 74.7 |  | 5 |  |  |  | 70.3 |
| 4 | | 69.2 | 68.4 | 68.2 | 69.6 |  | 6 |  |  |  | 18.5 |
| 5 | | 65.7 | 65.0 | 64.9 | 67.0 |  |  |  |  |  |  |

**Table S2** ^13^C-NMR (125 MHz) chemical shifts of saponins **10**−**13** in pyridine-*d*_5_

| C | **10** | | **11** | **12** | **13** |  | C | **10** | | **11** | **12** | **13** |
| --- | --- | --- | --- | --- | --- | --- | --- | --- | --- | --- | --- | --- |
| 1 | 38.7 | | 38.8 | 39.3 | 38.8 |  | 3 |  | | 72.4 | 72.5 | 82.9 |
| 2 | 25.9 | | 26.5 | 26.8 | 26.2 |  | 4 |  | | 73.9 | 74.1 | 73.0 |
| 3 | 82.1 | | 88.6 | 88.6 | 81.5 |  | 5 |  | | 69.8 | 69.8 | 69.8 |
| 4 | 43.4 | | 39.6 | 39.5 | 43.2 |  | 6 |  | | 18.6 | 18.6 | 18.4 |
| 5 | 47.8 | | 55.9 | 56.1 | 47.4 |  | Xyl |  | |  |  |  |
| 6 | 18.2 | | 18.5 | 18.4 | 18.1 |  | 1 |  | |  |  | 107.5 |
| 7 | 32.5 | | 33.1 | 34.6 | 32.7 |  | 2 |  | |  |  | 75.6 |
| 8 | 39.8 | | 39.8 | 41.2 | 39.8 |  | 3 |  | |  |  | 78.4 |
| 9 | 48.1 | | 48.1 | 50.8 | 48.1 |  | 4 |  | |  |  | 71.1 |
| 10 | 36.9 | | 37.0 | 37.1 | 36.8 |  | 5 |  | |  |  | 67.4 |
| 11 | 23.8 | | 23.7 | 21.2 | 23.8 |  | Glc I |  | |  |  |  |
| 12 | 122.8 | | 122.8 | 26.0 | 122.9 |  | 1 | 105.9 | |  | 106.4 |  |
| 13 | 144.0 | | 144.1 | 38.3 | 144.0 |  | 2 | 76.2 | |  | 75.4 |  |
| 14 | 42.2 | | 42.1 | 42.8 | 42.1 |  | 3 | 78.2 | |  | 78.5 |  |
| 15 | 28.4 | | 28.2 | 30.1 | 28.2 |  | 4 | 71.3 | |  | 71.2 |  |
| 16 | 23.3 | | 23.3 | 32.5 | 23.4 |  | 5 | 78.0 | |  | 78.7 |  |
| 17 | 47.1 | | 47.0 | 57.0 | 47.0 |  | 6 | 62.5 | |  | 62.5 |  |
| 18 | 41.7 | | 41.6 | 49.8 | 41.5 |  | 28-*O*-sugar | | |  |  |  |
| 19 | 46.1 | | 46.2 | 47.5 | 46.1 |  | Glc II | |  |  |  |  |
| 20 | 30.7 | | 30.8 | 150.9 | 30.7 |  | 1 | | 95.6 | 95.5 | 95.4 | 95.5 |
| 21 | 33.9 | | 33.9 | 30.8 | 33.9 |  | 2 | | 73.8 | 73.8 | 74.0 | 73.8 |
| 22 | 32.5 | | 32.5 | 37.1 | 32.4 |  | 3 | | 78.7 | 78.7 | 78.7 | 78.7 |
| 23 | 64.8 | | 28.1 | 28.1 | 64.2 |  | 4 | | 70.8 | 70.7 | 70.8 | 70.7 |
| 24 | 13.4 | | 17.1 | 16.3 | 13.9 |  | 5 | | 78.0 | 77.8 | 78.0 | 78.0 |
| 25 | 16.1 | | 15.6 | 16.7 | 16.1 |  | 6 | | 69.2 | 69.2 | 69.3 | 69.1 |
| 26 | 17.5 | | 17.4 | 16.4 | 17.5 |  | Glc III | |  |  |  |  |
| 27 | 26.2 | | 26.0 | 14.8 | 26.1 |  | 1 | | 104.8 | 104.7 | 105.0 | 104.8 |
| 28 | 176.5 | | 176.5 | 174.9 | 176.5 |  | 2 | | 75.3 | 75.2 | 75.3 | 75.3 |
| 29 | 33.0 | | 33.1 | 110.2 | 33.0 |  | 3 | | 76.4 | 76.4 | 76.4 | 76.4 |
| 30 | 23.6 | | 23.6 | 19.4 | 23.6 |  | 4 | | 78.2 | 78.2 | 78.1 | 78.2 |
| 3-*O*-sugar | | |  |  |  |  | 5 | | 77.1 | 77.1 | 77.2 | 77.1 |
| Ara | |  |  |  |  |  | 6 | | 61.2 | 61.1 | 61.2 | 61.2 |
| 1 | | 103.9 | 104.7 | 104.9 | 104.6 |  | Rha II | |  |  |  |  |
| 2 | | 81.3 | 75.8 | 76.3 | 75.3 |  | 1 | | 102.7 | 102.6 | 102.7 | 102.6 |
| 3 | | 73.6 | 73.8 | 74.0 | 74.9 |  | 2 | | 72.5 | 72.4 | 72.5 | 72.6 |
| 4 | | 68.2 | 68.6 | 79.6 | 69.5 |  | 3 | | 72.7 | 72.6 | 72.7 | 72.7 |
| 5 | | 64.9 | 65.0 | 64.5 | 66.4 |  | 4 | | 74.0 | 73.9 | 74.0 | 74.1 |
| Rha I | |  |  |  |  |  | 5 | | 70.2 | 70.2 | 70.3 | 70.2 |
| 1 | |  | 101.7 | 101.7 | 101.2 |  | 6 | | 18.5 | 18.4 | 18.5 | 18.5 |
| 2 | |  | 72.3 | 72.2 | 72.0 |  |  | |  |  |  |  |





**Figure**. **S1** Key NOESY and HMBC correlations for compound **2**.





**Figure** **S2** Key NOESY and HMBC correlations for compound **3**.

**

**

**Figure** **S3** Key NOESY and HMBC correlations for compound **4**.

**

**

**Figure** **S4** Key NOESY and HMBC correlations for compound **5**.
